# Comorbidities and Treatment Strategies in Bullous Pemphigoid: An Appraisal of the Existing Litterature

**DOI:** 10.3389/fmed.2018.00238

**Published:** 2018-09-04

**Authors:** Rikke Bech, Line Kibsgaard, Christian Vestergaard

**Affiliations:** Aarhus University Hospital, Aarhus, Denmark

**Keywords:** Bullous pemphigoid, cardio-vascular diseases, multiple sclerois, neurodegenarative diseases, mortality

## Abstract

Bullous Pemphigoid is an autoimmune skin blistering disease. It is caused by deposition of auto antibodies along the dermal-epidermal border leading to inflammation. The antibodies are directed against anchoring filaments in the epidermis, but these antigens are also present in the neurological tissues and this has led to speculation of an association between multiple sclerosis and bullous pemphigoid. Additionally recent epidemiological studies have pointed at an increased risk of cardio-vascualr diseases and an increased moratality among the patients with bullous pemphigoid. In this mini review we present the recent findings in this area and as well as the treatment strategies when comorbidities are taken into consideration.

## Introduction

Bullous pemphigoid (BP) is an autoimmune blistering, and often intensely itching, blistering skin disease affecting especially the elderly generation with an incidence of 14–43 pr. million. Children are rarely afflicted by this disease. The blisters are thick walled, as opposed to the pemphigus diseases. The disease may present in a non-bullous form, i.e., pre-bullous pemphigoid. The incidence of BP is increasing in western countries, and patients have an increased mortality compared to healthy controls with a 1 year mortality increased six to seven times and increased hazard ratio, HR = 2.4, for death as shown in a Danish registry study. The increased mortality within the first year after diagnosis may have a combined background. Firstly, in many guidelines systemic corticosteroids are listed as the first line treatment and many BP patients suffer from declining health and do have several co-morbidities including diabetes (type I and II), and ischemic heart disease as well as hypertension at the time of diagnosis. These are diseases that, when the patients are subjugated to treatment with systemic corticosteroids, increases the risk of death. Interestingly, the incidence of diabetes is increased among BP patients within the first year of diagnosis compared to an age and gender matched control group.

The pathogenesis of BP is dependent on IgG auto antibodies directed against the hemidesmosomes of the epidermal cells in the basement membrane. The antigens are the BP230 (BPAG1/Dystonin) and the BP180 (BPAG2/Type XVII Collagen) that upon binding induces inflammation in the skin and causes the formation of the blisters. Histologically a split on the dermo-epidermal border is seen along with an eosinophil dominated inflammation. Using direct immunofluorescence a linear band of IgG and/or C3 along the dermo-epidermal border can be demonstrated, as can circulating antibodies against BPAG1 or BPAG2.

The BPAG-1 is also expressed by the neurons and Schwann cells of the central nervous system and peripheral nervous system. This is believed to be the molecular background for the strong association between BP and multiple sclerosis (MS), OR = 10 in several studies. Other neurological diseases such as Parkinson disease and Alzheimer's disease also have an increased prevalence among patients diagnosed with BP, yet the pathogenetical pathways for this are not as clear as for MS.

In this short review we will go through the evidence and studies on the association between BP and cardiovascular as well as neurological diseases, in order to show that this group of dermatological patients do have need for a multidisciplinary approach and special care when it comes to treatment.

## Bullous pemphigoid and cardiovascular diseases

There is some evidence for an association between BP and cardiovascular diseases. Kibsgaard et al. described this in a retrospective consecutive case-series study of inpatients admitted to Department of Dermatology and Venerology in Aarhus, Denmark, between 2006 and 2013 (1). A total of 69 patients (70%) suffered from cardiovascular diseases (as determined by ICD10 diagnoses: I00 to I99) of which 39 were hypertension. The remaining cardiovascular diseases were congestive heart failure, arrhythmias, prior acute myocardial infarcts, dilated cardiomyopathy, and valvular diseases. The prevalence of cardiovascular diseases in the studied patients was larger than the known prevalence of cardiovascular diseases in the Danish background population. This may indicate an association between BP and cardiovascular disease. Similarly, Försti et al. found that cardiovascular diseases were the most common comorbidities among 198 cases with BP. This study was a retrospective database study of all cases of BP diagnosed at the Department of Dermatology, Oulu University Hospital, Finland, between 1985 and 2012 (2).

In contrast, Lee et al. found that hypertension was less frequent among patients with BP (48.5%) compared to the general Korean population (65.7%). The study of Lee et al. was a retrospective evaluation of 103 patients diagnosed with BP at the Chonnam National University Hospital in Gwangju, Korea, between 2006 and 2013 (3). Yet, hypertension was the most frequent comorbidity among the Korean BP patients with a univariable hazard ratio (HR) of 1.23(0.67–2.25). However, the HR for hypertension was non-significant (*p*-value 0.505) when compared to the background population (3). This might be a result of the BP patients older age. Mean age of the BP patients in this study was 74.4 years at time of bp diagnosis.

Similarly, Banishahemi et al. found that hypertension is the most frequent comorbidity (22.9%) among patients with BP (4), and that the prevalence of hypertension was lower in the studied BP patients compared to the Iranian population older than 55 years of age. In this descriptive cross-sectional study of 122 Iranian patients the mean age of the BP patients was 69 years.

In a Danish population based cohort study of 3,500 BP patients a significantly increased OR (1.7) of hypertension at the time of diagnosis was also found (5). Yet, when following up the risk of developing hypertension was the same as the age and sex matched control population (HR = 1.0) and even significantly decreased (HR = 0.8) if the first year of observation was excluded thus not including patients who had died within the first observational year. This may be due to patients diagnosed with BP had more contacts to the health care system and would thus be treated for other diseases that could lead to hypertension. Alternatively, the results could indicate that BP may be the endstage of several systemic disease, or as it has been suggested; that certain drugs may increase the risk of BP (6).

The possible association between BP and cardiovascular diseases could be a result of inhibited fibrinolysis and coagulation activation in patients with BP. In an observational study of 20 BP patients compared to 20 age and sex matched healthy subjects levels of plasminogen activator inhibitor type 1 (PAI-1) antigen, tissue plasminogen activator (t-PA) antigen, fibrin fragment d-dimer and prothrombin fragment were compared (7). An increase in PAI-1 in patients with active BP but also an increase of t-PA, fibrin fragment d-dimer and prothrombin were found, indicating inhibited fibrinolysis and a coagulation activation in the patients with BP. Marzano et al. also found that during remission after treatment with corticosteroids levels of PAI-1 and fibrin fragment d-dimer were reduced, thus reducing the inhibition of fibrinolysis and decreasing the trombotic risk.

Another possible explanation of the association between BP and cardiovascular disease could be that the BP antigen (BP230) is expressed in cardiac muscle. Steiner-Champliaud showed that the BP230 is expressed in primate cardiac muscle cells (8). Also, Andrä et al. found disruption of the intercalated discs of the myocardium in BP230 deficient mice (9). Boyer et al. found signs of cardiac stress in BP230 deficient mice (10).

## Bullous pemphigoid and neurological disease

The association between BP and neurodegenerative diseases is well-described (11, 12). There is evidence that having a neurodegenerative disease increases the risk for developing BP and that patients with neurodegenerative diseases have a higher standardized mortality rate. The molecular link between BP and neurodegenerative diseases is hypothesized to be autoantibodies against the antigens BP180 and BP230 expressed in neurons as well as the basement membrane of human skin. However, only few immune pathological studies have confirmed this theory. Yet, the question is: Which came first, the hen or the egg, hence the neurodegenerative disease or bullous pemphigoid?

Försti et al. studied 198 Finnish cases with BP and found that the most common comorbidities were cardiovascular diseases (76.3%) and neurodegenerative diseases (40.9%) (13). Teixeira et al. made a case control study with 77 cases and 176 controls (14). At least one neurological disease was present in 55.8% (43) of bullous pemphigoid cases before the diagnosis of BP compared with 20.5% (36) of controls (OR 5.36, 95% CI 2.97–9.66). Jeon et al. retrospectively evaluated 103 patients diagnosed with BP between 2006 and 2013 in Korea and found that among patients with BP the prevalence of dementia, and Parkinson's disease was higher than in the general population (3). Kibsgaard et al. found in a population-based cohort study of BP patients that the second most frequent comorbidity was neurologic disorders, comprising multiple sclerosis (5).

**Table 1:** Bullous pemphigoid and comorbidities.

	**Study design**	**Cases (*N*)**	**Mean age (years)**	**Female: male ratio**	**Controls (*N*)**	**Confounders**	Cardiovascular diseases^*^(%)	**Neurological comorbidities (%)**	**Comparative measures**
Kibsgaard et al. (1) (Denmark)	Case-series (retrospective)	98	78 (SD ± 10)	1.7	NA	Adjusted for age and sex	70	Stroke: 12.2, dementia: 17.3 PD: 4, MS: 1	NA
Banishahemi et al. (4) (Iran)	Retrospective descriptive study	122	69	NA	NA	NA	NA	Epilepsy: 0.8 PD: 1.6	NA
Casas-de-la-Asunción et al. (18) (Spain)	Observational, retrospective, case-control	54	80.8	1.5	108	Adjusted for age and sex	NA	NA	**OR** of dementia: 4.6 (2.1–10.1), PD: 5.9 (1.8–19.7), CVD: 3.1 (1.2–7.9)
Gambichler et al. (21) (Germany)	Retrospective database study AND experimental study	161	85/78 (±ND)	2.7 (+ND) 2.6 (–ND)	NA	NA	NA	Dementia: 48.2, Stroke: 17.6, PD: 14.1	NA
Gornowicz-Porowska et al. (19) (Poland)	Case-series (retrospective)	82	78.5/74 (±ND)	0.8 (+ND) 2.6 (–ND)	NA	NA	NA	24	Pearson X2 test used to check for relationship between ND development and the BP180/360 anti IgG level
Kalinska-Bienias et al. (25) (Poland)	Case-series (retrospective)	205	76.2	0.6	NA	NA	38	Dementia: 22.4, stroke: 13.7 PD: 4.9, depression:4.4	NA
Khosravani et al.(15) (Iran/USA)	Cross-sectional case-control study	87	Cases: 64.2 Controls: 60.7	Cases: 1.1 Controls: 2.1	184	NA	NA	Cerebrovascular disease: 8 Dementia: 16.8	NA
Marzano et al. (7) (Italy)	Follow up study	20	76	1	20	NA	NA	NA	NA
Ren et al., 2017 (USA)	Cross sectional	2.105	75.9 (primary) 77.5 (secondary)	1.6	1.5	NA	CHF: 2.7, AFLI: 2	NA	**OR** Vascular dementia: 1.7, PD: 1.8
Tarazona et al. (16) (Brazil)	Cross sectional	25	73.9	2.6	NA	NA	NA	NA	NA
Brick et al. (17) (USA)	Case-control & matched cohort design	87	77.5	Cases: 1.4 Controls: 1.3	261	Adjustet for age (±2 years) and sex	NA	**Case group**: 17 **Control group**: 11	**OR** of dementia: 9.0 (2.4–33.2), PD: 7.2 (0.6- infinity), MS: 3.0 (0.19–48.0) & CVD: 1.8 (0.4–7.5) **HR** of dementia 1.3 (0.6–2.6), PD: 8.56 (1.6–47.3), CVD: 2.5 (0.9–7.1)
Försti et al. (13) (Finland)	Case-control & matched cohort design	4.524	NA	Cases: 1.5 Controls: 1.2	66.138	Adjusted for age and sex	NA	**Cases**: CNS: 41, Psyciatruc: 10.4 **Controls**: CNS: 16, Psyc: 7	**OR** of Alzheimers disease: 2.6 (2.3–3.0), PD: 2.4 (2.0–2.9), MS: 5.87 (3.93–8.53) **HR** of Alzheimers disease: 1.2 (1.1–1.4), PD: 1.1 (0.8–1.5), MS: 0.7 (0.1–5.0), schizophrenia 1.4 (0.6–3.4)
Kibsgaard et al. (5) (Denmark)	Case-control & matched cohort design	3.281	76.5 (SD± 12.6)	Cases: 1.3 Controls: 1.3	32.213	Adjusted for age and sex	Case group: 57.6 Control group: 45.6	**Case group**:34.5 **Control group**: 22.4	**OR** of Alzheimers: 2.6 (1.8–3.5), PD: 4.2 (3.1–5.8), MS: 9.7 (6.0–15.6) and hypertension: 2.0 (1.8–2.2) **HR** of Alzheimers: 0.83 (0.6–1.1), Parkinsonism: 0.7 (0.4–1.2) MS: 9.4 (4.9–18.0) and hypertension: 1.1 (1.0–1.2)

* *Within the studied BP population*.

In a case control study a cerebrovascular accident (CVA) was the most common neurological disease which was seen in 7 patients (8.0%) in the case group and 4 patients (2.1%) in the control group (*p* = 0.022) (15), also dementia was significantly increased 16.8 vs. 1.0% (*p* = 0.008).

In a Brazilian cohort of patients with BP a significantly higher prevalence of neurological and/or psychiatric diseases was found (16). Especially cerebrovascular accident (CVA) and dementia were over-represented. Brick et al. found in their case control study an association of BP with neurologic disorders such as dementia and Parkinson disease (17). In a Spanish case-control study patients with BP were found to have a higher frequency of neurologic conditions (18).

The hypothetical pathogenetic association between BP and neurological disease has been questioned by Gornowicz-Porowska et al. finding insignificant differences in the autoantibody (BP180 and BP230) levels of patients with BP with and without neurological disease (19). Similarly, Ali et al. found no significant correlation between the transcripts of BPAG1a in the central nervous system and a diverse spectrum of neurological disorders related with BP (20).

Whether BP precedes neurological diseases or vice versa, fosters an ongoing discussion. Gambichler et al. found in their database of inpatients with BP a significantly increased frequency of neurological diseases in BP patients (21). They also studied brain tissue of mammalians treated with serum from nine patients with BP and elevated BP180 autoantibodies. These studies showed that raised BP180 titres and blood eosinophils were independent predictors for the presence of neurological disease in the patients with BP. However, the experimental data did not support previous results indicating that specific binding of BP180 antibodies in neuronal tissue plays a pathogenetic role in neurological disease. Taghipour et al. found that patients with BP and neurological disease exhibit an immune response to both BP180 and BP230, and thus hypothesize that both antigens may be exposed following a neurological insult followed by generation of an immune response in terms of BP (22).

In many patients with the co-existence of BP and neurological disease, the onset of neurological disease precedes the onset of BP with many years. We have considered this apparent paradox, and we hypothesize that the time lag between neurological disease and the onset of BP might be a result of the gradual exposure of autoantigens (BP180 and BP230) as the neurological disease progresses. The titres of anti-BP180/230 might increase as these antigens are exposed in the neurological tissues. Thereby increasing the risk of developing symptoms in other tissues containing BP180/230, for example skin and mucous membranes.

## Treatment of bullous pemphigoid

BP has traditionally been treated with topical steroids supplemented with systemic corticosteroid treatment if necessary. This is reflected in the British Association of Dermatologists' (BAD) 2012 guidelines for the management of BP (23). Superpotent topical corticosteroids are recommended as first-line treatment in localized and moderate disease, and in generalized BP the only validated systemic treatment is oral prednisolone. Yet, there is broad consensus among dermatologists to the use of other immunomodulating treatments in order to minimize the accumulated dose of systemic corticosteroids and the consequential detrimental side effects.

In the retrospective Finnish study by Försti et al., it was found that polypharmacy was very common in patients with BP, and the higher the number of drugs, the greater the mortality (24). Thus, the mortality for BP in Finland is 7.6-fold that of a reference population, due to malignancies and polypharmacy.

Kibsgaard et al. found in their retrospective consecutive case-series study of 98 BP patients a significant difference in admission time in favor of patients treated with low dose prednisolone (<45 mg/day) (*p* = 0.02) vs. patients treated with high dose prednisolone (>45 mg/day) (1). This may be due to the fact that patients treated with high doses of systemic glucocorticoid were initially suffering from more severe disease. However, this association could not be shown in the following sub-analysis. In contrast, rate of remission and relapse, median duration of systemic corticosteroid and immune modulating treatments, and total treatment duration were independent of the dichotomized initial doses of systemic glucocorticoids. There was no information on development of comorbidities in the two groups.

Similarly, Kalinska-Bienias et al. found that prednisone in moderate dose (0.5 mg kg−1) in monotherapy was an independent risk factor of fatal prognosis in the 1st year of follow-up, assessed by multivariate analysis (25). Patients treated with prednisone in monotherapy were in this study associated with almost a two-time increased risk of mortality. A weak correlation was found (in univariate analysis only) that the patients who received tetracycline plus nicotinamide showed decreased mortality within the 1st year of follow-up.

These results are in line with the co-morbidities of cardio-vascular diseases and diabetes as described above, and since prednisolone may further accentuate these diseases and increase the mortality as can be seen in the studies by systemic corticosteroid may be responsible for the increased mortality during the first year after diagnosis.

In order to minimize the daily as well as the cumulative dose of systemic corticosteroid, so-called steroid sparing immune modulating drugs can be used to treat bullous pemphigoid. The immune modulating agents can be used in combination with topical steroids and/or systemic corticosteroids or alone.

A pragmatic, multicenter, randomized controlled trial by Williams et al. underpins that Doxycycline is non-inferior to standard treatment with oral prednisolone for short-term blister control in bullous pemphigoid and significantly safer in the long-term (26). The role, dosing and duration of tetracycline (e.g., plus nicotinamide) treatment must, however, be further established in future randomized controlled trials.

Sticherlin et al. found that Dapsone appeared to have a moderately higher corticosteroid-sparing potential than azathioprine (27, 28). Yet, due to the lower than intended number of patients, the results of the primary and secondary endpoints were not or only barely significant. The combination regimen of either drug with oral methylprednisolone was associated with a relatively low 1-year mortality. This was a prospective, multicentre, randomized, non-blinded clinical trial comparing the efficacy and safety of two parallel groups of patients with BP treated with oral methylprednisolone in combination with either azathioprine or dapsone.

Azathioprine is also used as a steroid sparing agent against bullous pemphigoid. The effect and side effects of Azathioprine in the treatment of bullous pemphigoid was compared to Mycophenolate Mofetil, both in combination with oral prednisolone, in a prospective, multicenter, randomized, non-blinded clinical trial (29) showing equal efficacy.

Methotrexate is a commonly used as a steroid sparing agent for BP as found by Kibsgaard et al. considered equally effective as Azathioprine for the treatment of bullous pemphigoid. However, this has never been studied in a randomized controlled clinical trial.

Other prednisolone sparing immune modulating agents include Cyclosporine, Rituximab (28, 30, 31), intravenous immunoglobulin (32), Cyclophosphamide (33), and plasmapheresis (34, 35). Some of these treatments can be used in combination (36).

In our opinion oral prednisolone is an effective treatment with profound evidence of effectiveness in the treatment of severe BP. However, increased morbidity is strongly associated to especially long term use of oral prednisolone. Therefore, we recommend the immediate institution of steroid sparing treatment when the clinical diagnosis of BP has been pitched. Histological diagnosis should be awaited. For patients with diabetes and in the very old patients, we prefer to omit oral prednisolone and instead use highly potent topical steroids once or twice daily for some weeks and to treat the patients with oral Doxycycline (26, 37) or Dapsone (27, 38, 39). BP patients usually experience effect of Doxycycline or Dapsone, respectively, within 3–4 weeks.

A new publication by Quick et al. elucidates BP antigens as potential targets in future therapy of patients with BP (11).

## Conclusion

BP is associated with increased risk for cardiovascular disease and neurological diseases. Whether the pathogenetic processes in BP predisposes to cardiovascular diseases and neurological diseases or vice versa has not been fully established yet.

The most well-established association is between multiple sclerosis and BP, which may also be explained biologically through the formation of antibodies against BP180 and BP230 which are expressed in the skin and in the neurological tissue. Thus biology and epidemiology is closely related.

In terms of cardiovascular disease the most prominent is hypertension which in many, but not all studies, seems to be associated with BP. As a consequence, there may also be an association with other cardiovascular diseases including valvular diseases.

Systemic corticosteroids are effective in the treatment of BP. However, the use of systemic corticosteroids prolongs admission time for the patients and increases the BP patients morbidity and mortality. Thus systemic corticosteroids have detrimental effects on hypertension and cardiovascular diseases and may as such bias the perception of the association between these diseases.

Although there are few studies on the use of non-corticosteroid immunemodulating treatment of BP, the findings of shorter admission time, and increased mortality within the first year of treatment and the fact that prednisolone monotherapy may double mortality leads us to conclude that non-corticsteroid treatment should be instituted as early as possible after the diagnosis of BP.

## Author contributions

All authors listed have made a substantial, direct and intellectual contribution to the work, and approved it for publication.

### Conflict of interest statement

The authors declare that the research was conducted in the absence of any commercial or financial relationships that could be construed as a potential conflict of interest. The reviewer CDS and handling Editor declared their shared affiliation.

## References

[1] Kibsgaard L Bay B Deleuran M Vestergaard C. A retrospective consecutive case-series study on the effect of systemic treatment, length of admission time, and co-morbidities in 98 bullous pemphigoid patients admitted to a tertiary centre. Acta Derm Venereol. (2015) 95:307–11. 10.2340/00015555-1925 24979241

[2] Försti AK Huilaja L Schmidt E Tasanen K. Neurological and psychiatric associations in bullous pemphigoid—more than skin deep? Exp Dermatol. (2017) 26:1228–34. 10.1111/exd.13401 28677172

[3] Lee JH Kim SC. Mortality of patients with bullous pemphigoid in Korea. J Am Acad Dermatol. (2014) 71:676–83. 10.1016/j.jaad.2014.05.006 24930586

[4] Banihashemi M Zabolinejad N Vahabi S Razavi HS. Survey of bullous pemphigoid disease in northern iran. Int J Dermatol. (2015) 54:1246–9. 10.1111/ijd.12619 25783773

[5] Kibsgaard L Rasmussen M Lamberg A Deleuran M Olesen AB Vestergaard C. Increased frequency of multiple sclerosis among patients with bullous pemphigoid: a population-based cohort study on comorbidities anchored around the diagnosis of bullous pemphigoid. Br J Dermatol. (2017) 176:1486–91. 10.1111/bjd.15405 28235244

[6] Joly P Roujeau JC Benichou J Picard C Dreno B Delaporte E (2002). A comparison of oral and topical corticosteroids in patients with bullous pemphigoid. N Engl J Med. 346:321–7. 10.1056/NEJMoa011592 11821508

[7] Marzano AV Tedeschi A Polloni I Crosti C Cugno M. Prothrombotic state and impaired fibrinolysis in bullous pemphigoid, the most frequent autoimmune blistering disease. Clin Exp Immunol. (2013) 171:76–81. 10.1111/j.1365-2249.2012.04674.x 23199326 PMC3530098

[8] Steiner-Champliaud MF Schneider Y Favre B Paulhe F Praetzel-Wunder S Faulkner G. BPAG1 Isoform-b: complex distribution pattern in striated and heart muscle and association with plectin and α-actinin. Expe Cell Res. (2010) 316:297–313. 10.1016/j.yexcr.2009.11.010 19932097

[9] Andrä K Lassmann H Bittner R Shorny S Fässler R Propst F. Targeted inactivation of plectin reveals essential function in maintaining the integrity of skin, muscle, and heart cytoarchitecture. Genes Dev. (1997) 11:43–56. 10.1101/gad.11.23.3143 9389647 PMC316746

[10] Boyer JG Bhanot K Kothary R Boudreau-Larivière C. Hearts of Dystonia musculorum mice display normal morphological and histological features but show signs of cardiac stress. PLoS ONE (2010) 5:e9465. 10.1371/journal.pone.0009465 20209123 PMC2830884

[11] Quick QA. Microtubule-actin crosslinking factor 1 and plakins as therapeutic drug targets. Int J Mol Sci. (2018) 19:E368. 10.3390/ijms19020368 29373494 PMC5855590

[12] Ren Z Hsu DY Brieva J Silverberg NB Langan SM Silverberg JI. Hospitalization, inpatient burden and comorbidities associated with bullous pemphigoid in the U.S.A. Br J Dermatol. (2017) 176:87–99. 10.1111/bjd.14821 27343837

[13] Försti AK Jokelainen J Ansakorpi H Seppänen A Majamaa K Timonen M. Psychiatric and neurological disorders are associated with bullous pemphigoid - a nationwide finnish care register study. Sci Rep. (2016) 6:37125. 10.1038/srep37125 27845416 PMC5109264

[14] Teixeira VB Cabral R Brites MM Vieira R Figueiredo A. Bullous pemphigoid and comorbidities: a case-control study in portuguese patients. Anais Bras Dermatol. (2014) 89:274–8. 10.1590/abd1806-4841.20142516 24770504 PMC4008058

[15] Khosravani S Handjani F Alimohammadi R Saki N. Frequency of neurological disorders in bullous pemphigoid patients: a cross-sectional study. Int Sch Res Notices (2017) 2017:6053267. 10.1155/2017/6053267 28630891 PMC5463116

[16] Tarazona MJ Mota AN Gripp AC Unterstell N Bressan AL. Bullous pemphigoid and neurological disease: statistics from a dermatology service. Anais Bras Dermatol. (2015) 90:280–2. 10.1590/abd1806-4841.20153334 25831008 PMC4371687

[17] Brick KE Weaver CH Savica R Lohse CM Pittelkow MR Boeve BF. A population-based study of the association between bullous pemphigoid and neurologic disorders. J Am Acad Dermatol. (2014) 71:1191–7. 10.1016/j.jaad.2014.07.052 25174542 PMC4326088

[18] Casas-de-la-Asunción E Ruano-Ruiz J Rodríguez-Martín AM Vélez García-Nieto A Moreno-Giménez JC. Association between bullous pemphigoid and neurologic diseases: a case-control study. Actas Dermosifiliogr. (2014) 105:860–5. 10.1016/j.adengl.2014.09.010 25004905

[19] Gornowicz-Porowska J Seraszek-Jaros A Bowszyc-Dmochowska M Kaczmarek E Pietkiewicz P Bartkiewicz P. Analysis of the autoimmune response against BP180 and BP230 in ethnic poles with neurodegenerative disorders and bullous pemphigoid. Central Eur J Immunol. (2017) 42:85–90. 10.5114/ceji.2017.67322 28680335 PMC5470618

[20] Ali A Hu L Zhao F Qiu W Wang P Ma X. BPAG1, a distinctive role in skin and neurological diseases. Semin Cell Dev Biol. (2017) 69:34–9. 10.1016/j.semcdb.2017.06.005 28627382

[21] Gambichler T Segert H Höxtermann S Schmitz L Altmeyer P Teegen B. Neurological disorders in patients with bullous pemphigoid: clinical and experimental investigations. J Eur Acad Dermatol Venereol. (2015) 29:1758–62. 10.1111/jdv.12995 25651418

[22] Taghipour K Chi CC Bhogal B Groves RW Venning V Wojnarowska F. Immunopathological characteristics of patients with bullous pemphigoid and neurological disease. J Eur Acad Dermatol Venereol. (2014) 28:569–73. 10.1111/jdv.12136 23530989

[23] Venning VA Taghipour K Mohd Mustapa MF Highet AS, Kirtschig G. British association of dermatologists' guidelines for the management of bullous pemphigoid 2012. Br J Dermatol. (2012) 167:1200–14. 10.1111/bjd.12072 23121204

[24] Försti A Jokelainen J Timonen M Tasanen K Risk of death in bullous pemphigoid: a retrospective database study in finland. Acta Dermato Venereol. (2014) 17:758–61. 10.2340/00015555-2347 26806363

[25] Kalinska-Bienias A Lukowska-Smorawska K Jagielski P Kowalewski C Wozniak K. Mortality in bullous pemphigoid and prognostic factors in 1st and 3rd year of follow-up in specialized centre in poland. Arch Dermatol Res. (2017) 309:709–19. 10.1007/s00403-017-1772-x 28852833

[26] Williams HC Wojnarowska F Kirtschig G Mason J Godec TR Schmidt E. Doxycycline versus prednisolone as an initial treatment strategy for bullous pemphigoid: a pragmatic, non-inferiority, randomised controlled trial. Lancet (2017) 389:1630–8. 10.1016/S0140-6736(17)30560-3 28279484 PMC5400809

[27] Sticherling M Franke A Aberer E Gläser R Hertl M Pfeiffer C Rzany B. An open, multicentre, randomized clinical study in patients with bullous pemphigoid comparing methylprednisolone and azathioprine with methylprednisolone and dapsone. Br J Dermatol. (2017) 177:1299–305. 10.1111/bjd.15649 28494097

[28] Cho YT Chu CY Wang LF. First-line combination therapy with rituximab and corticosteroids provides a high complete remission rate in moderate-to-severe bullous pemphigoid. Br J Dermatol. (2015) 173:302–4. 10.1111/bjd.13633 25529394

[29] Beissert S Werfel T Frieling U Böhm M Sticherling M Stadler R Zillikens D A comparison of oral methylprednisolone plus azathioprine or mycophenolate mofetil for the treatment of bullous pemphigoid. Arch Dermatol. (2007) 143:1536–42. 10.1001/archderm.143.12.1536 18087004

[30] Ahmed AR Shetty S Kaveri S Spigelman ZS. Treatment of recalcitrant bullous pemphigoid (BP) with a novel protocol: a retrospective study with a 6-year follow-up. J Am Acad Dermatol. (2016) 74:700.e3–8.e3. 10.1016/j.jaad.2015.11.030 26851830

[31] Shen AL Lin HL Lin HC Tseng YF Hsu C Chou CY. Increased risk of bullous pemphigoid after first-ever stroke: a population-based study. Neurodegener Dis. (2017) 17:166–70. 10.1159/000469710 28467996

[32] Amagai M Ikeda S Hashimoto T Mizuashi M Fujisawa A Ihn H Matsuzaki Y. A randomized double-blind trial of intravenous immunoglobulin for bullous pemphigoid. J Dermatol Sci. (2017) 85:77–84. 10.1016/j.jdermsci.2016.11.003 27876358

[33] Gual A Iranzo P Mascarõ JM. Treatment of bullous pemphigoid with low-dose oral cyclophosphamide: a case series of 20 patients. J Eur Acad Dermatol Venereol. (2014) 28:814–8. 10.1111/jdv.12155 23581830

[34] Kaneda H Shimizu M Yachie A Toyama M Municipal H Dear E Bullous pemphigoid successfully treated with a combination therapy of plasmapheresis followed by intravenous high dose immunoglobulin. Ther Apheresis Dial. (2017) 21:421–3. 10.1111/1744-9987.12536 28378368

[35] Chang B Tholpady A Huang RS Nedelcu E Bai Y. Clinical and serological responses following plasmapheresis in bullous pemphigoid: two case reports and a review of the literature. Blood Transfus. (2014) 12:269–75. 10.2450/2014.0222-13 24931844 PMC4039711

[36] Ridpath AV Rzepka PV Shearer SM Scrape SR Olencki TE Kaffenberger BH. Novel use of combination therapeutic plasma exchange and rituximab in the treatment of nivolumab-induced bullous pemphigoid. Int J Dermatol. (2018). [Epub ahead of print]. 10.1111/ijd.13970 29624655

[37] Chalmers JR Wojnarowska F Kirtschig G Mason J Childs M Whitham D. A randomised controlled trial to compare the safety, effectiveness and cost-effectiveness of doxycycline (200 Mg/Day) with that of oral prednisolone (0.5 Mg/Kg/Day) for0(BLISTER) trial. Health Technol Assess. (2017) 21:1–90. 10.3310/hta21100 28406394 PMC5402219

[38] Piette EW Werth VP. Dapsone in the management of autoimmune bullous diseases. Immunol Allergy Clin North Am. (2012) 29:561–4. 10.1016/j.det.2011.06.018 22560144 PMC3928009

[39] Grcan HM Ahmed AR Efficacy of dapsone in the treatment of pemphigus and pemphigoid: analysis of current data. Am J Clin Dermatol. (2009) 10:383–396. 10.2165/11310740-000000000-00000 19824739

